# Comprehensive Analyses of PANoptosome with Potential Implications in Cancer Prognosis and Immunotherapy

**DOI:** 10.1007/s10528-024-10687-8

**Published:** 2024-03-04

**Authors:** Yonghua Cai, Heng Xiao, Qixiong Zhou, Jie Lin, Xianqiu Liang, Wei Xu, Yongfu Cao, Xian Zhang, Hai Wang

**Affiliations:** 1 Department of Neurosurgery, Nanfang Hospital, Southern Medical University, Guangzhou, 510515 Guangdong People’s Republic of China; 2 Southern Medical School, No. 1023, South Shatai Road, Baiyun District, Guangzhou, 510515 Guangdong China; 3 Department of Neurosurgery, Key Laboratory of Biological Targeting Diagnosis, Therapy and Rehabilitation of Guangdong Higher Education Institutes, The Fifth Affiliated Hospital, Guangzhou Medical University, Guangzhou, China

**Keywords:** PANoptosome, PANoptosis, Anti-tumor immunity, Immunotherapy

## Abstract

Cell death resistance significantly contributes to poor therapeutic outcomes in various cancers. PANoptosis, a unique inflammatory programmed cell death (PCD) pathway activated by specific triggers and regulated by the PANoptosome, possesses key features of apoptosis, pyroptosis, and necroptosis, but these cannot be accounted for by any of the three PCD pathways alone. While existing studies on PANoptosis have predominantly centered on infectious and inflammatory diseases, its role in cancer malignancy has been understudied. In this comprehensive investigation, we conducted pan-cancer analyses of PANoptosome component genes across 33 cancer types. We characterized the genetic, epigenetic, and transcriptomic landscapes, and introduced a PANoptosome-related potential index (PANo-RPI) for evaluating the intrinsic PANoptosome assembly potential in cancers. Our findings unveil PANo-RPI as a prognostic factor in numerous cancers, including KIRC, LGG, and PAAD. Crucially, we established a significant correlation between PANo-RPI and tumor immune responses, as well as the infiltration of diverse lymphoid and myeloid cell subsets across nearly all cancer types. Moreover, a high PANo-RPI was consistently associated with improved immunotherapy response and efficacy, as evidenced by re-analysis of multiple immunotherapy cohorts. In conclusion, our study suggests that targeting PANoptosome components and modulating PANoptosis may hold tremendous therapeutic potential in the context of cancer.

**Supplementary Information:**

The online version contains supplementary material available at 10.1007/s10528-024-10687-8.

## Introduction

Cancer is defined as a group of diseases characterized by abnormal cell growth associated with pathological manifestations as well as significant morbidity and mortality globally (Choi et al. 2010; Bray et al. 2018). As cancers exhibit dysregulated cell death and inflammatory responses (Hanahan and Weinberg 2000, 2011; Green and Evan 2002), many current therapeutic approaches focus on inducing cancer cell death preferentially (Bernier et al. 2004; Carneiro and El-Deiry 2020). However, evasion of cell death, especially apoptosis, is one of the major mechanisms of primary and adaptive therapeutic resistance in tumors, which in turn leads to poor therapeutic efficacy (Hanahan and Weinberg 2000, 2011; Letai 2008). PANoptosis, a unique inflammatory programmed cell death (PCD) pathway activated by specific triggers and regulated by the PANoptosome, possesses key features of apoptosis, pyroptosis, and necroptosis, but cannot be accounted for by any of the three PCD pathways alone (Nguyen and Kanneganti 2021; Place et al. 2021; Wang and Kanneganti 2021; Gullett et al. 2022; Liu et al. 2022). PANoptosome acts as a molecular scaffold for the contemporaneous engagement of key apoptotic, pyroptotic, and necroptotic machinery. It is a multifaceted multiprotein complex whose main components include proteins such as RIPK1, RIPK3, NLRP3, CASP1, CASP6, CASP8, FADD, and PYCARD (Samir et al. 2020; Zheng and Kanneganti 2020; Zheng et al. 2020; Christgen et al. 2020, 2021; Jiang et al. 2021a, b; Nguyen and Kanneganti 2021; Place et al. 2021; Wang and Kanneganti 2021; Gullett et al. 2022; Liu et al. 2022). Some triggers (including influenza A virus, vesicular stomatitis virus, *Listeria monocytogenes*, and *Salmonella enterica serovar Typhimurium*) initiate the assembly of PANoptosome by activating specific sensors, such as ZBP1, and then promote the activation of downstream PCD executioners, including CASP3/CASP7-mediated apoptosis, GSDMD/GSDME-mediated pyroptosis, and RIPK1/MLKL-mediated necroptosis (Malireddi et al. 2019, 2020; Christgen et al. 2020, 2021; Zheng et al. 2020; Jiang et al. 2021a, b; Place et al. 2021; Wang and Kanneganti 2021; Gullett et al. 2022).

Recently, Karki et al. found that the synergism of tumor necrosis factor α (TNF-α) and interferon γ (IFN-γ) triggers caspase-8/FADD-mediated PANoptosis by activating the JAK/STAT1/IRF1 axis in SARS-CoV-2 Infection (Karki et al. 2021a). Subsequently, they identified that ZBP1-mediated PANoptosis disrupts interferon therapeutic efficacy during coronavirus infection, including SARS-CoV-2 and mouse hepatitis virus (MHV) (Karki et al. 2022). Most previous studies on PANoptosis have focused on infectious diseases; however, emerging studies have shown that it is closely related to cancers. For instance, Karki et al. in 2020 identified that interferon regulatory factor 1 (IRF1) as an upstream regulator of PANoptosis to restrict tumorigenesis in colitis-associated colorectal cancer (Karki et al. 2020). In 2021, adenosine deaminase acting on RNA 1 (ADAR1) could restrict ZBP1-mediated PANoptosis activated by combining interferons (IFNs) and nuclear export inhibitors (such as leptomycin B and Selinexor) to promote tumorigenesis (Karki et al. 2021b). Subsequently, Malireddi et al. found that IFN-γ and TNF-α together could trigger PANoptosis of tumor cells and suppress tumor growth in colon and lung cancers, melanoma, and leukemia (Malireddi et al. 2021). In addition, Lin et al. in 2022 demonstrated that phosphorylated cysteine desulfurase (NFS1) could prevent PANoptosis from weakening oxaliplatin-based chemosensitivity in colorectal cancer (Lin et al. 2022). However, the complex correlation between PANoptosis and molecular characteristics, clinical features, and treatment strategies in cancers warrants further exploration.

In this study, we focused on PANoptosome core components and comprehensively explored their genomic, epigenomic, and transcriptomic characteristics across 33 cancers. We found that the expression of PANoptosome components was significantly associated with distinct genomic and epigenetic events. Subsequently, we developed a scoring system, the PANoptosome-related potential index (PANo-RPI), to investigate the potential association between PANopotosis and immune signatures. PANo-RPI was highly correlated with many immune response-related pathways and the tumor infiltration of immune cells. Furthermore, the association between PANo-RPI and immunotherapy response was elucidated by reanalyzing multiple immune checkpoint inhibitor (ICI) therapy cohorts, demonstrating that high PANo-RPI was correlated with better immunotherapy response and efficacy. Based on these findings, we hypothesized that small-molecule drugs could potentially activate the assembly of the PANoptosome and induce PANoptosis. This integrated analysis provides a rich resource for understanding PANoptosome biology and preliminarily unveils the potential application of the PANoptosome as a predictive biomarker for immunotherapy response.

## Materials and Methods

### Data Preparation and Processing

The uniformly normalized pan-cancer dataset, which integrates the Cancer Genome Atlas (TCGA, http://cancergenome.nih.gov/) and Genotype-Tissue Expression (GTEx, http://commonfund.nih.gov/GTEx/) databases, and the corresponding clinical data were downloaded from the UCSC Xena (https://xenabrowser.net/datapages/). The gene expression matrices and clinical data of the immunotherapy cohorts (PRJEB23709 (Gide et al. 2019), GSE91061 (Riaz et al. 2017), GSE100797 (Lauss et al. 2017), and GSE35640 (Ulloa-Montoya et al. 2013)) were downloaded from the BioProject and GEO datasets at NCBI (https://www.ncbi.nlm.nih.gov/).

### Bioinformatics Analysis and Online Analysis Platforms

Single nucleotide variation (SNV), copy number variation (CNV), and methylation analyses were conducted using the Gene Set Cancer Analysis ( http://bioinfo.life.hust.edu.cn/GSCA/#/) platform (Liu et al. 2018). The upstream TFs and miRNAs of PANoptosome genes were predicted using the RegNetwork website (https://regnetworkweb.org/) (Liu et al. 2015), and the TFs-miRNAs-PANoptosome genes network was constructed using the Cytoscape software (Shannon et al. 2003), which was downloaded from https://cytoscape.org/. The differentially expressed genes (DEGs) between tumor and non-tumor tissues was performed via the “limma” R package (Ritchie et al. 2015). The PANo-RPI was calculated using the ssGSEA method with the “gsva” R package (Hanzelmann et al. 2013). Pathway activity based on the “hallmark gene sets” from the molecular signatures database (MsigDB, https://www.gsea-msigdb.org/gsea/msigdb) (Liberzon et al. 2015) was assessed using the GSVA method with the “gsva” R package (Hanzelmann et al. 2013). Certain biological process signatures, including antigen processing machinery, immune checkpoints, epithelial-mesenchymal transition (EMT), etc., were evaluated according to the protocol described by Mariathasan et al. (2018). The immune cell infiltrations were analyzed using the ESTIMATE algorithm (Yoshihara et al. 2013) and ImmuCellAI database (http://bioinfo.life.hust.edu.cn/web/ImmuCellAI/) (Miao et al. 2020). The correlation between PANoptosome genes and the sensitivity of small-molecule drugs from the genomics of drug sensitivity in cancer (GDSC, https://www.cancerrxgene.org/) (Miao et al. 2020) and cancer therapeutics response portal (CTRP, https://portals.broadinstitute.org/ctrp/) (Basu et al. 2013; Seashore-Ludlow et al. 2015; Rees et al. 2016) were evaluated using GSCA (Liu et al. 2018). The 3D coordinates of proteins were downloaded from the PDB (http://www.rcsb.org/pdb/home/home.do) (ww 2019), the structures of small-molecule drugs were retrieved from PubChem Compound (https://pubchem.ncbi.nlm.nih.gov/) (Wang et al. 2017), and the binding affinities and modes was analyzed through Autodock Vina 1.2.2, which is an in silico protein–ligand docking software.

### Statistical Analysis and Visualization

R 4.0.2 software (https://www.r-project.org/) was used for statistical analysis and data visualization. The student’s t-test was performed to assess differences, and Pearson or Spearman tests were used to evaluate correlations between continuous variables. The R “ggplot2” package was used to visualize the data. Statistical significance was defined as a two-tailed *P*-value < 0.05. *P < 0.05; **P < 0.01; ***P < 0.001, ****P < 0.0001.

## Results

### The Genetic, Epigenetic, and Transcriptional Characteristics of PANoptosome Component Genes

**Fig. 1:**
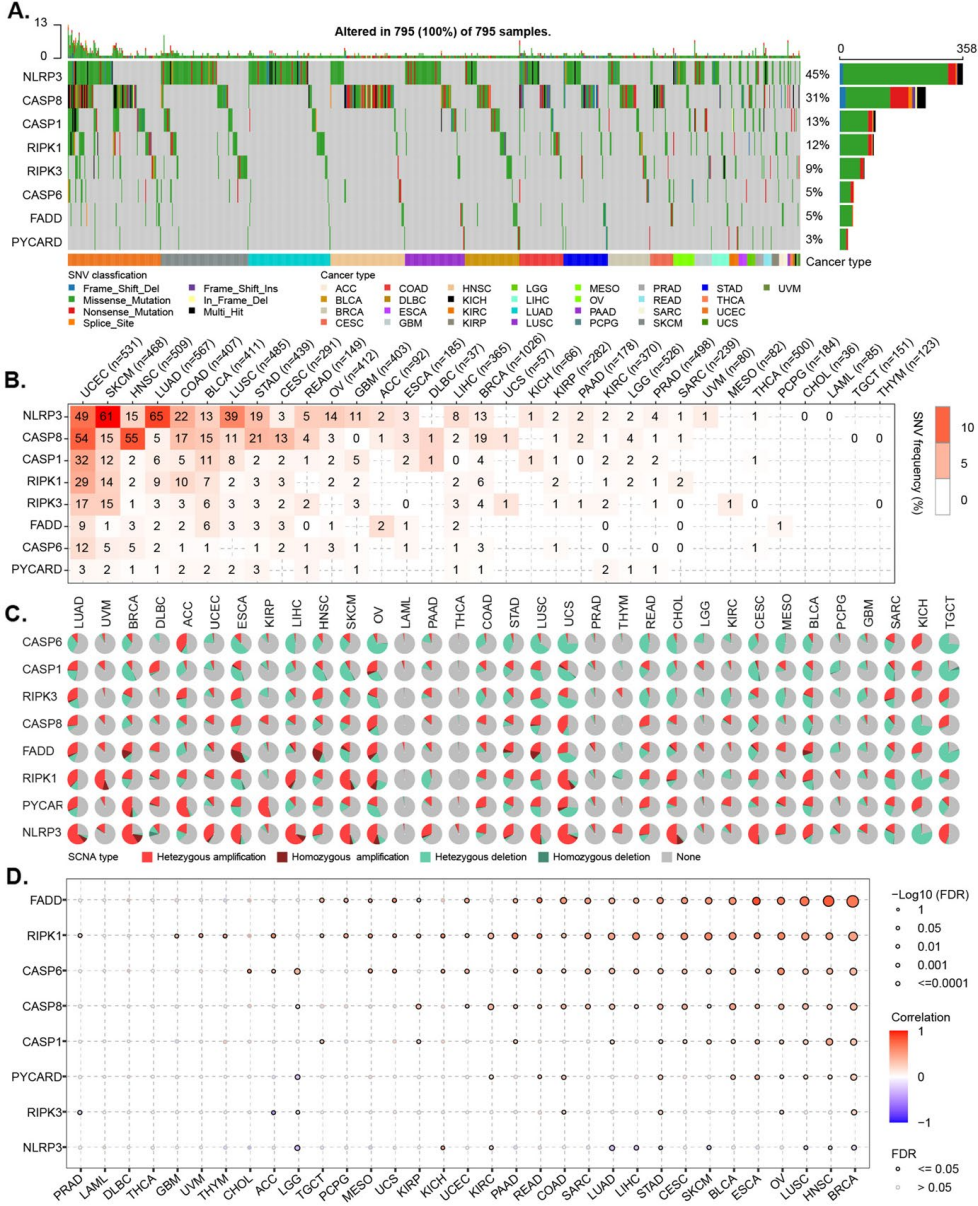
Genetic Alterations in PANoptosome-Related Core Genes. **A** The genomic landscape of SNVs in PANoptosome-related genes in cancers. The frequency of alterations in eight PANoptosome-related genes is presented. Only samples (*n* = 795) with genomic alterations are shown. The alteration rates per gene are indicated on the right labels. **B** The distribution of SNV frequencies. **C** An overview of the proportion of different CNV types of PANoptosis-related genes. **D** The correlation between CNV levels and mRNA expression for PANoptosis-related genes

**Fig. 2:**
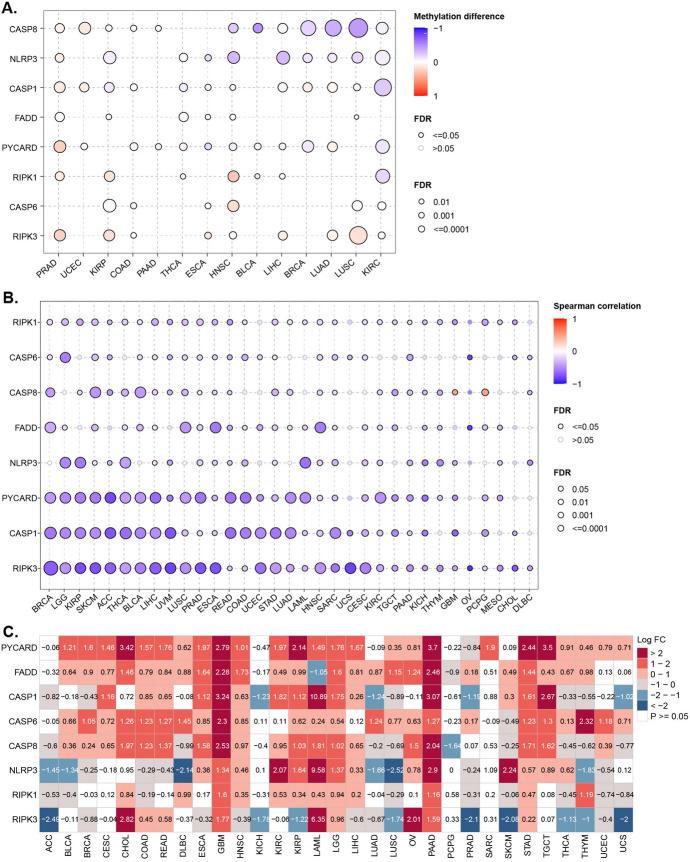
Aberrant expression of PANoptosome-related core genes. **A** The variations in the methylation of PANoptosome-related genes between tumor and normal samples. **B** The correlation between methylation and mRNA expression of PANoptosome-related genes. **C** The results of the DEGs analysis of PANoptosome-related genes between tumor and normal tissues

To study the genomic characteristics of PANoptosome core components in cancers, we first investigated SNV across 33 cancer types (Table S1). The SNV frequency of PANoptosome component genes ranged from 3 to 45% (Fig. 1A). The Fig. S1A summarizes the SNV classes of PANoptosome components. We found that the most common variant classification was missense mutation, the most common variant type was single nucleotide polymorphism (SNP), and the most common SNV class was C > T. Among them, the SNV frequency of NLRP3 (45%) and CASP8 (31%) was the highest whereas that of the PYCARD (3%) was the lowest. At the genomic level, mutations may be the main cause of dysregulation of the PANoptosome components in these cancers (Figs. 1A and S1A). By further analyzing the SNV profile of the PANoptosome gene set, we observed that certain cancer types displayed more alterations compared to others. For example, UCEC, SKCM, HNSC, and LUAD showed alterations in all eight genes, whereas THYM, TGCT, LAML, and CHOL did not show any alterations (Fig. 1B). Additionally, we found that certain components showed significantly distinct frequencies in different cancer types. Specifically, the SNV frequency of NLRP3 was high in LUAD **(**65%) and SKCM (61%), whereas that of CASP8 was high in HNSC (55%) and UCEC (54%) (Fig. 1B). To identify CNV of PANoptosome component genes, we used the CNV data from TCGA database. The pie chart (Fig. 1C) displays the CNV percentage of the PANoptosome components in each cancer. The Fig. S1B, C provide the profile of heterozygous and homozygous CNV of PANoptosome components, indicating that heterozygous deletion and amplification are the main types of CNV. Correlation analysis demonstrated that the expression of most of the PANoptosome components was positively correlated with CNV levels in many cancers (such as BRCA, HNSC, LUSC, and OV), especially for FADD, RIPK1, and CASP6. However, there was a negative correlation between CNV and NLRP3 in the LGG, LUAD, LIHC, STAD, SKCM, LUSC, HNSC, and BRCA groups (Fig. 1D). Subsequently, we investigated the methylation of PANoptosome components to identify the features of epigenetic regulation. As shown in Fig. 2A, the methylation patterns of PANoptosome components in different cancers were significantly heterogeneous. In particular, CASP8 and NLRP3 were hypomethylated in many cancers, whereas RIPK3 was hypermethylated. Interestingly, most PANoptosome components were hypomethylated in KIRC and hypermethylated in PRAD (Fig. 2A). Surprisingly, correlation analysis showed that most of the expression levels of the PANoptosome components were negatively correlated with their methylation levels, and only CASP8 in GBM and PCPG displayed a positive correlation between gene expression and methylation (Fig. 2B). These results imply that somatic and epigenetic alterations of PANoptosome components may be involved in altering their abnormal expression in diverse cancer contexts, which might play a vital role in cancer progression. Since transcription factors (TFs) and microRNAs (miRNAs) can regulate the mRNA expression of protein-coding genes, we predicted the potential TFs and miRNAs and constructed a TFs-miRNAs-PANoptosome components network (Fig. S2). Finally, DEG analysis was performed to compare the mRNA expression of PANoptosome components between cancer and non-cancer tissues. These PANoptosome components, but not RIPK3, showed an abnormally high expression in cancers. Moreover, all of these genes were significantly upregulated in GBM, LGG, PAAD, and STAD (Fig. 2C).

### The Development of Pan-Cancer PANo-RPI and Its Clinical Relevance

**Fig. 3:**
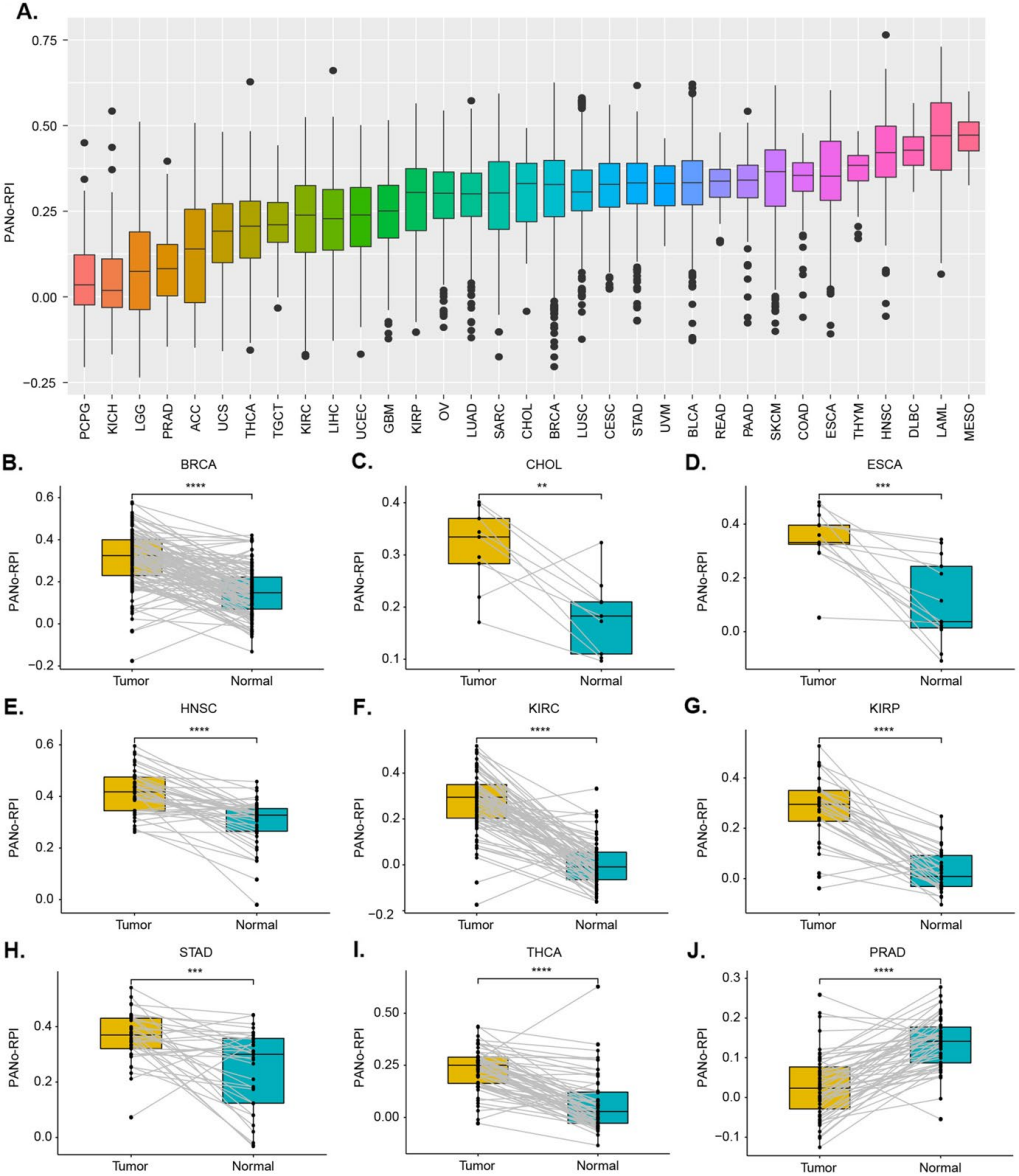
The development of PANo-RPI in cancers. **A** The PANo-RPI levels in 33 different cancer types. **B–J** Comparative analysis of PANo-RPI between tumor tissues and adjacent normal tissues is presented for specific cancers, including **B** BRCA, **C** CHOL, **D** ESCA, **E** HNSC, **F** KIRC, **G** KIRP, **H** STAD, **I** THCA, and **J** PRAD

To further explore the potential clinical relevance of PANoptosis from an overall perspective, we performed a single-sample gene set enrichment analysis (ssGSEA) to develop the computational model PANo-RPI for the assessment of the PANoptosome assembly potential based on the expression of eight core PANoptosome component genes, including RIPK1, RIPK3, NLRP3, CASP1, CASP6, CASP8, FADD, and PYCARD. All cancers showed a wide range of PANo-RPIs, and all had some individuals with high PANo-RPIs (Fig. 3A). By comparing the levels of PANo-RPI, significant heterogeneity was found across 33 cancers. Specifically, The PANo-RPI was distinctly high in MESO, LAML, DLBC, HNSC, and THYM, whereas it was demonstrably low in PCPG and KICH (Fig. 3A). Additionally, we found that the PANo-RPI of tumors was higher than normal tissues in BRCA, CHOL, ESCA, HNSC, KIRC, KIRP, STAD, THCA, BLCA, and CESC (Fig. S3A–J) but lower in PRAD, COAD, and LUAD (Fig. S3K–M). Furthermore, we compared the PANo-RPI levels between cancerous and adjacent non-cancerous specimens. The comparison showed that PANo-RPI levels were increased in BRCA, CHOL, ESCA, HNSC, KIRC, KIRP, STAD, and THCA (Fig. 3B–I) but decreased in PRAD (Fig. 3J). These findings suggest that the PANoptosome or PANoptosis has distinct relationships with different cancers during tumorigenesis and progression.

**Fig. 4:**
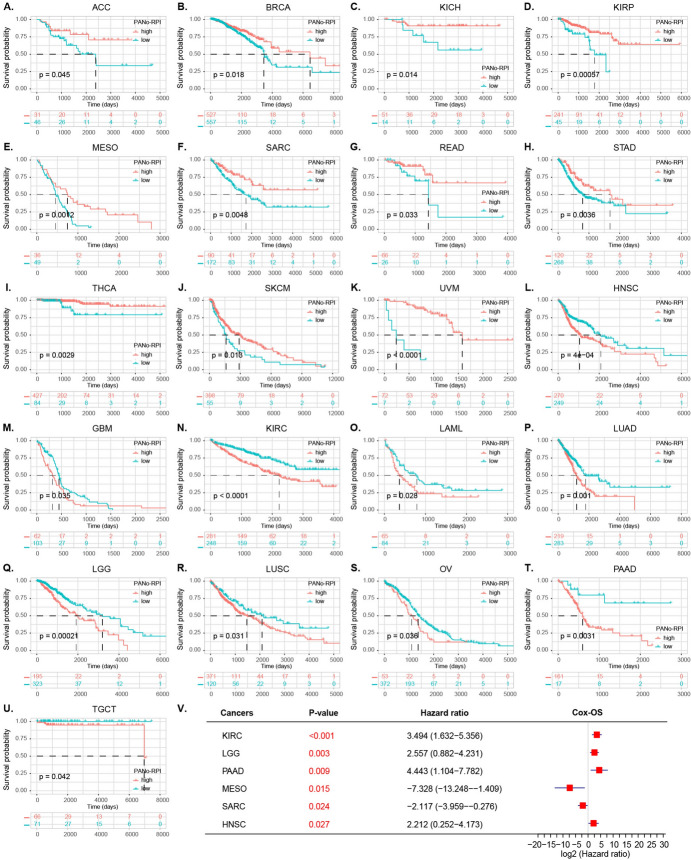
Prognostic Value of PANo-RPI and Its Regulators. **A**–**U** Kaplan–Meier OS curves in patients with **A** ACC, **B** BRCA, **C** KICH, **D** KIRP, **E** MESO, **F** SARC, **G** READ, **H** STAD, **I** THCA, **J** SKCM, **K** UVM, **L** HNSC, **M** GBM, **N** KIRC, **O** LAML, **P** LUAD, **Q** LGG, **R** LUSC, **S** OV, **T** PAAD and **U** TGCT according to high and low PANo-RPI. **V** Forest plot of univariate Cox regression analyses of OS for PANo-RPI in cancers

**Fig. 5:**
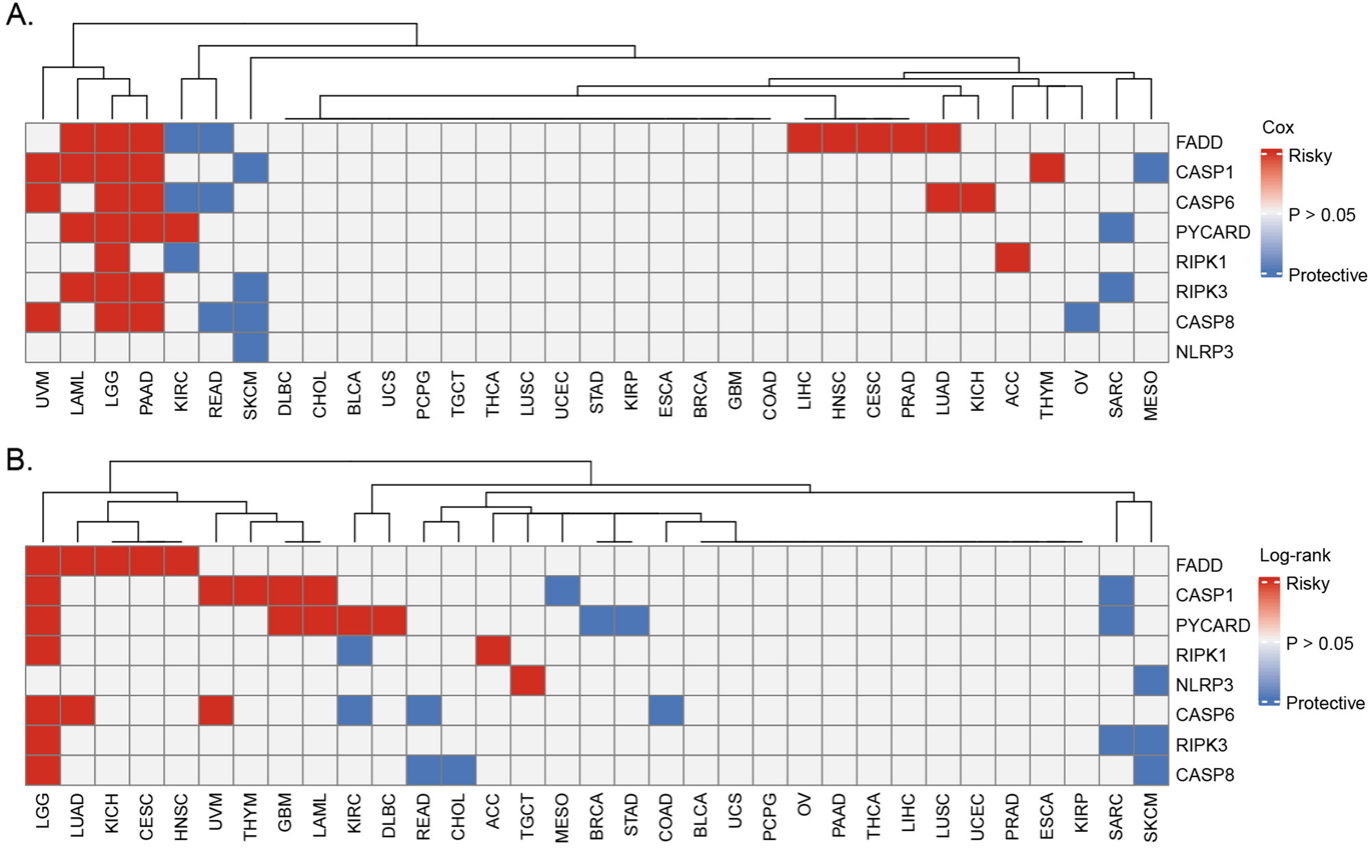
Heatmap depicting the results of the univariate Cox regression analyses **A** and Log-rank test **B** for OS of each PANoptosome-related genes in 33 cancer types. Red indicates risky factors, and blue indicates protective factors

To investigate the association between the PANoptosome and cancer survival, overall survival (OS) and progression-free interval (PFI) analyses were conducted based on PANo-RPI levels. Kaplan–Meier and univariate Cox regression analyses were used to explore the correlation between PANo-RPI with OS and PFI in all 33 cancers. Patients with high PANo-RPI had longer OS than those with low PANo-RPI in ACC, BRCA, KICH, KIRP, MESO, SARC, READ, STAD, THCA, SKCM, and UVM (Fig. 4A–K). However, contrasting findings were observed with HNSC, GBM, KIRC, LAML, LUAD, LGG, LUSC, OV, PAAD, and TGCT (Fig. 4L–U). The forest plot of COX analysis (Fig. 4V) indicated that the PANo-RPI was an unfavorable prognostic factor for the OS in KIRC (HR = 3.494, 95%CI 1.632−5.356, *P* < 0.001), LGG (HR = 2.557, 95%CI 0.882−4.231, *P* = 0.003), PAAD (HR = 4.443, 95%CI 1.104−7.782, *P* = 0.009), HNSC (HR = 2.212, 95%CI 0.252−4.173, *P* = 0.027), whereas it was a favorable prognostic factor in MESO (HR = − 7.238, 95%CI − 13.248–− 1.409, *P* = 0.015) and SARC (HR = − 2.117, 95%CI − 3.959–− 0.276, *P* = 0.024). As for the PFI, patients with high PANo-RPI had longer progression-free survival time than those with low PANo-RPI in ACC, BRCA, UVM, ESCA, DLBC, UCEC, and UCS (Fig. S4A–G). Contrasting findings were observed for KIRC, LGG, LUAD, LUSC, PAAD, PCPG, PRAD, TGCT, THYM, GBM, and HNSC (Fig. S4H–R). The forest plot of COX analysis (Fig. S4S) implied that PANo-RPI was an unfavorable prognostic factor in KIRC (HR = 4.681, 95%CI 2.717–6.644, *P* < 0.001), PAAD (HR = 5.607, 95%CI 2.413–8.801, *P* < 0.001), LGG (HR = 2.057, 95%CI 0.745−3.370, *P* = 0.002), PCPG (HR = 5.811, 95%CI 1.128−10.495, *P* = 0.015), GBM (HR = 2.481, 95% 0.457−4.505, *P* = 0.016), and THYM (HR = 13.701, 95% = 0.374−27.029, *P* = 0.044) but a favorable prognostic factor in UVM (HR = − 11.704, 95%CI − 19.081−− 4.326, *P* = 0.002) and BRCA (HR = − 2.546, 95%CI − 4.330–− 0.763, *P* = 0.005). Finally, we performed univariate Cox regression analysis to investigate the prognostic value of each PANoptosome component gene across 33 cancers. We found that some PANoptosome genes (such as CASP6 and CASP8) were risk factors for UVM, LAML, LGG, and PAAD and protective factors for SKCM and READ (Fig. 5A, B). Detailed statistics for the tests are shown in Table S2.

### The Correlation of PANo-RPI with Cancer Immunity

**Fig. 6:**
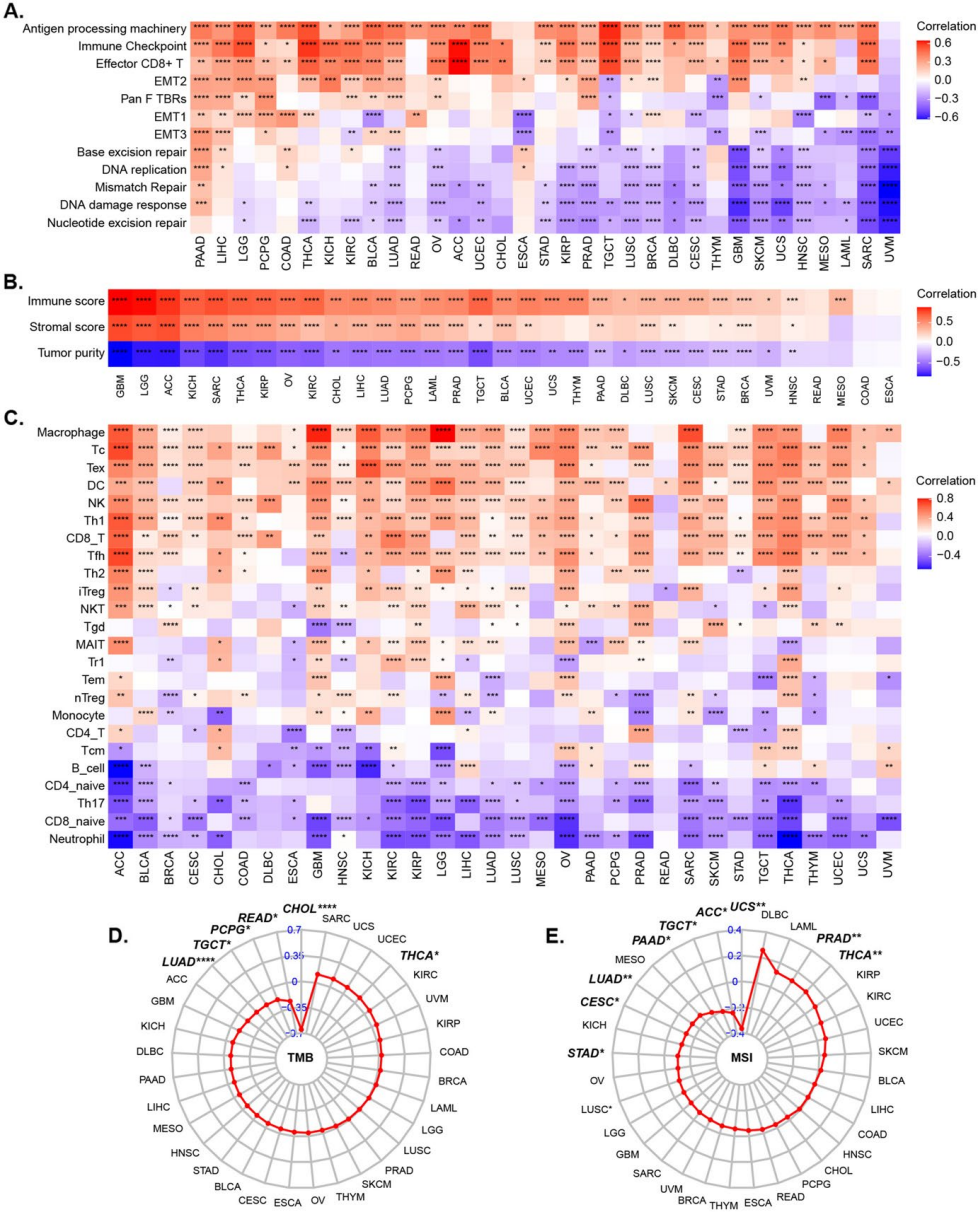
The correlation between PANo-RPI and cancer immunity. **A** Correlation between PANo-RPI and the enrichment scores of gene sets developed by Mariathasan et al. **B** Correlation between PANo-RPI and immune scores, stromal scores, and tumor purity. **C** Correlation between PANo-RPI and the proportions of the immune cell subsets. **D**, **E** Correlation between PANo-RPI and **D** TMB and **E** MSI

To explore the biological interpretations underlying the PANoptosome and PANoptosis, we first conducted gene set variation analysis (GSVA) based on hallmark pathway gene sets. As shown in Fig. S5 and Table S3, PANo-RPI was typically positive correlated with the GSVA scores of pathways such as allograft rejection, interferon gamma response, IL6/JAK/STAT3 signaling, inflammatory response, complement, interferon alpha response, p53 pathway, IL2/STAT5 signaling, apoptosis, reactive oxygen species pathway, and TNF alpha signaling via NFKB, whereas it was negatively correlated with pathways such as protein secretion, spermatogenesis, mitotic spindle, androgen response, hedgehog signaling, pancreas beta cells, and so on. Subsequently, we investigated the correlation between PANo-RPI and certain biological process signatures referred to in the gene sets provided by Mariathasan et al. (Mariathasan et al. 2018) in cancers. Strikingly, we observed that PANo-RPI had a significant positive correlation with immune-related signatures (including antigen processing machinery, immune checkpoints, and effector CD8+T cells), whereas it showed a strong negative correlation with DNA damage and repair pathways (such as nucleotide excision repair, DNA damage response, mismatch repair, DNA replication, and base excision repair) in almost all human cancers (Fig. 6A and Table S4). These results implied that the PANoptosome and PANoptosis were highly associated with cancer cell fate, survival or death, and tumor immune remodeling. Given that the composition of non-tumor cells significantly influences the immune status of the tumor microenvironment (Oike et al. 2018; Kunk et al. 2021; Hasan et al. 2022), we further explored the associations of PANo-RPI with tumor immune cells and stromal cell infiltration using the ESTIMATE method. As shown in Fig. 6B and Table S5, PANo-RPI was positively correlated with the immune score (in 30 out of 33 cancers, Fig. S6A) and stromal score (in 24 out of 33 cancers, Fig. S6B) but negatively correlated with tumor purity (in 29 out of 33 cancers, Fig. S6C). Additionally, we found that PANo-RPI was positively correlated with most chemokines (Fig. S7A) and chemokine receptors (Fig. S7B). These results indicate that the activation of PANoptosome assembly may promote the infiltration of non-tumor cells, especially immune cells. To further infer the recruitment of certain tumor-infiltrating immune cells, the proportions of the immune cell subsets were calculated using ImmuCellAI. Subsequently, we performed Pearson’s correlation analysis and found that PANo-RPI was significantly associated with many immune cell subsets (Fig. 6C and Table S6). For lymphoid lineage cells, PANo-RPI was positively correlated with the infiltration of CD8+T cells, cytotoxic T cells (Tc), exhausted T cells (Tex), helper T-cell 1 (Th1), helper T-cell 2 (Th2), follicular helper T-cell (Tfh), regulatory T cells (Tregs), NK cells, and NKT cells, but negatively correlated with that of naïve CD8+T cells, helper T-cell 17 (Th17), naïve CD4+T cells, and B cells; for myeloid lineage cells, PANo-RPI had a significantly positive correlation with the infiltration of macrophages and dendritic cells (DCs), whereas it was negatively correlated with neutrophils (Fig. 6C). Tumor mutation burden (TMB) and microsatellite instability (MSI) are essential biomarkers in tumor microenvironment (TME), and are considered valid response indicators of immunotherapies in many kinds of cancers (Luchini et al. 2019). Therefore, the association of TMB/MSI with PANo-RPI was evaluated. The radar charts (Fig. 6D, E) showed that PANo-RPI was positively correlated with TMB scores in THCA but negatively correlated with CHOL, READ, PCPG, TGCT, and LUAD (Table S7). PANo-RPI was positively correlated with MSI scores of PRAD and THCA and negatively correlated with those of UCS, ACC, TGCT, PAAD, LUAD, CESC, and STAD (Table S8). The aforementioned results indicate that the PANoptosome, in addition to being related to cell death, is highly likely to be involved in mediating immune signaling regulation and anti-cancer immune responses.

### The Potential of PANo-RPI in Predicting Immunotherapy Response and Efficacy

**Fig. 7:**
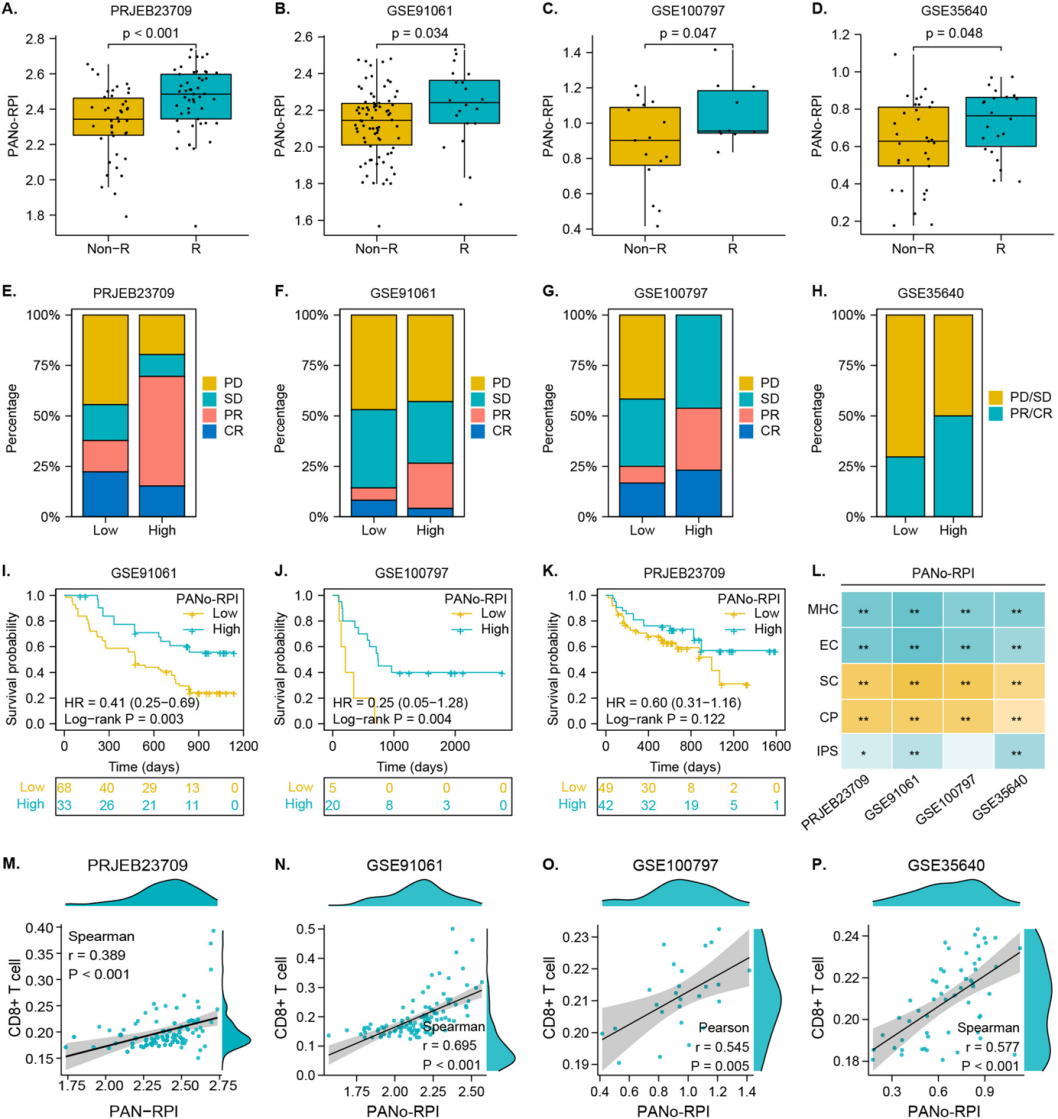
Potential of PANo-RPI to predict immunotherapy response. **A**–**D** Comparison of PANo-RPI levels between responders (R) and non-responders (NR) in immunotherapy cohorts from **A** PRJEB23709, **B** GSE91061, **C** GSE100797 and **D** GSE35640 datasets. Rate of clinical response (complete response (CR), partial response (PR), stable disease (SD), and progressive disease (PD)) to immunotherapies in high or low PANo-RPI groups in the **E** PRJEB23709, **F** GSE91061, **G** GSE100797 and **H** GSE35640 cohorts. **I**–**K** Kaplan–Meier curves for low and high PANo-RPI groups in **I** GSE91061, **J** GSE100797 and **K** GSE35640 cohorts. **L** Correlation heatmap between PANo-RPI and MHC, EC, CP, SC, and IPS scores in the four immunotherapy cohorts. **M**–**P** Correlation scatterplots between CD8+T-cell infiltrations and PANo-RPI in **M** PRJEB23709, **N** GSE91061, **O** GSE100797, and **P** GSE35640 cohorts

Considering the distinct association between PANo-RPI and anti-cancer immunity, we assessed whether PANo-RPI was associated with the response and efficacy of cancer immunotherapies. Four immunotherapy datasets (PRJEB23709, GSE91061, GSE100797, and GSE35640) were used in this study. We evaluated the PANo-RPI of non-responders and responders and found that the PANo-RPI of responders was higher than that of non-responders in all four immunotherapy cohorts (*P* < 0.05) (Fig. 7A–D). Subsequently, we separated patients into high and low PANo-RPI groups using the corresponding median level and counted the occupation ratios of immunotherapeutic responses (complete response, CR; partial response, PR, stable disease, SD; and progressive disease, PD) in the two groups. The stacked bar plots show that the objective tumor response (CR+PR) was significantly improved in the high PANo-RPI group (Fig. 7E–H). Additionally, survival analysis indicated that after immunotherapies, patients with high PANo-RPI had a significantly longer survival time than those with low PANo-RPI in GSE91061 (Log-rank *P* = 0.003) and GSE100797 (Log-rank *P* = 0.004) (Fig. 7I, J). Although not statistically significant, the PRJEB23709 cohort showed a similar trend (Fig. 7K). Furthermore, we calculated the aggregated scores of antigen processing (MHC), effector cells (EC), suppressor cells (SC), and CP (checkpoints) using Immunophenogram (Charoentong et al. 2017). The results showed that PANo-RPI was positively correlated with MHC and EC but negatively correlated with SC and CP (Fig. 7L). Additionally, the immunophenoscore (IPS) is considered a superior predictor of the ICI response (Charoentong et al. 2017). It was significantly correlated with PANo-RPI in three out of the four immunotherapy cohorts in our study (Fig. 7L). These results imply that PANo-RPI has remarkable potential for predicting immunotherapy response and efficacy in many cancers. Since the number and functions of CD8+T cells are major factors associated with immunotherapeutic activity, we assessed the association between PANo-RPI and CD8+T-cell infiltration. As expected, correlation analysis demonstrated that PANo-RPI had a significant positive correlation with the CD8+T-cell proportion in PRJEB23709 (Spearman: *r* = 0.389, *P* < 0.001), GSE91061 (Spearman: *r* = 0.695, *P* < 0.001), GSE100797 (Pearson: *r* = 0.545, *P* = 0.005), and GSE35640 (Spearman: *r* = 0.577, *P* < 0.001) (Fig. 7M–P). Taken together, the aforementioned analyses revealed that the PANoptosome may potentiate the immune response and efficacy by remodeling the tumor immune microenvironment (TIME), such as recruiting immune cells and maintaining the differentiation and functions of CD8+T cells.

### Potential Small-Molecule Drugs Based on PANoptosome Components

**Fig. 8:**
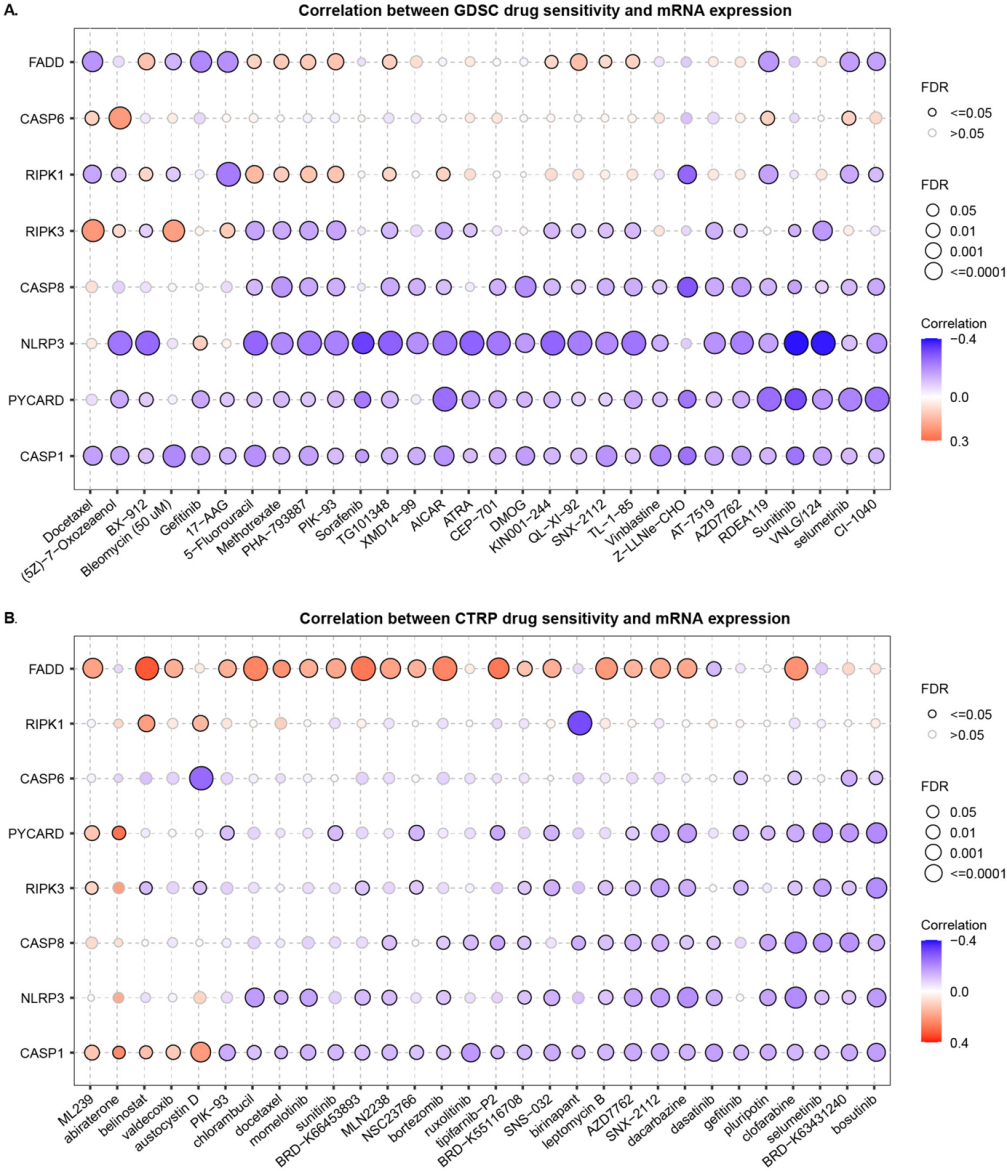
Potential small-molecule drugs based on PANoptosome components. **A**, **B** Correlation heatmaps depicting the relationships between the expression PANoptosis-related genes and the sensitivity of small-molecule drugs in GDSC and CTRP databases

**Fig. 9:**
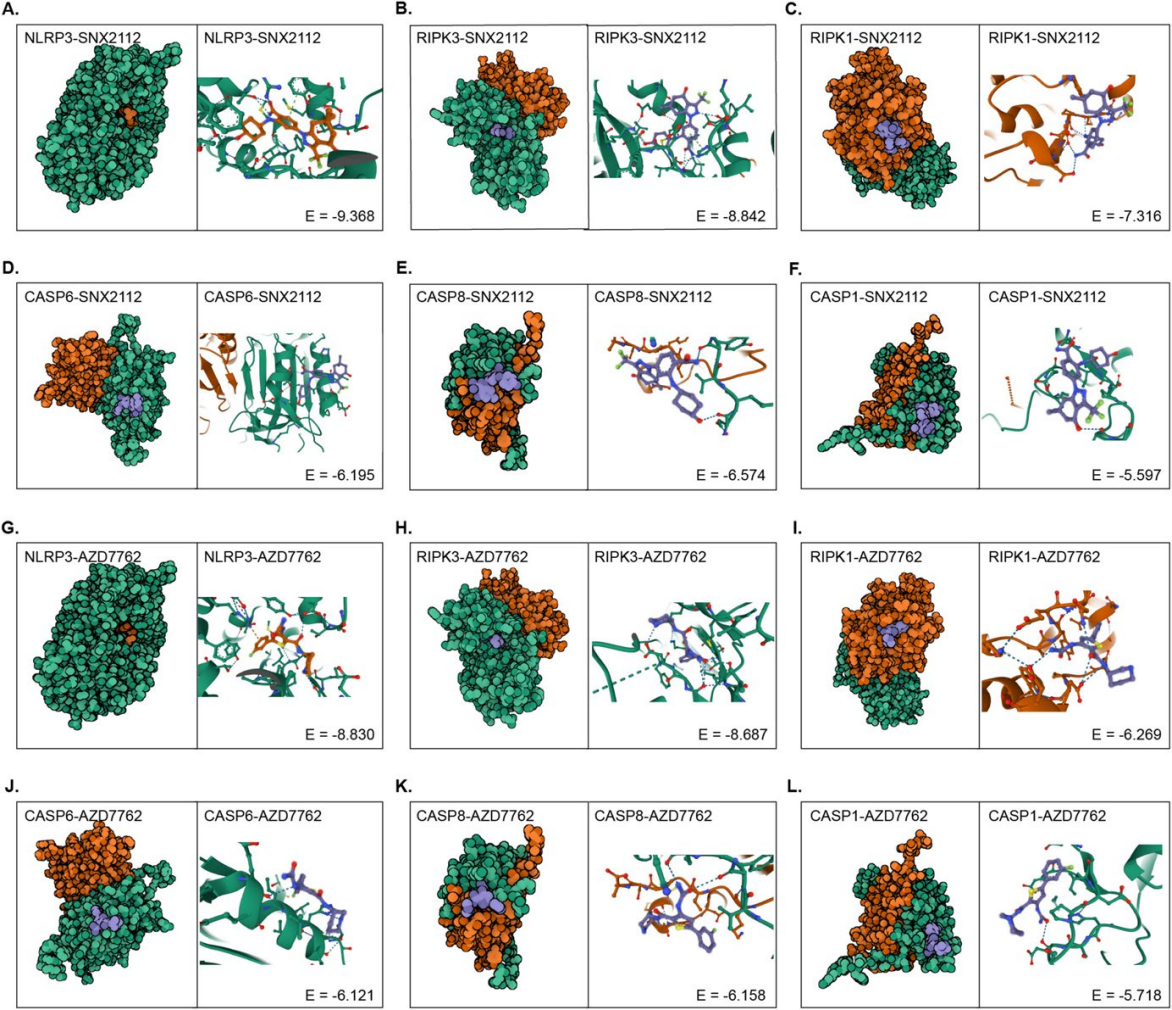
Molecular docking of small-molecule drugs and PANoptosome Components. **A**–**F** Molecular docking simulations of SNX-2112 with **A** NLRP3, **B** RIPK3, **C** RIPK1, **D** CASP6, **E** CASP8 and **F** CASP1. **G**–**L** Molecular docking simulations of AZD7762 with **G** NLRP3, **H** RIPK3, **I** RIPK1, **J** CASP6, **K** CASP8 and **L** CASP1. The left cartoons depict the crystal structures of small-molecule compounds and their respective targets. The three-dimensional structures on the right illustrate the binding pockets. Hydrogen bonds are represented by blue dashed lines, and Pi stacking is indicated by orange or green bonds

Strategies that can activate PANoptosome assembly and induce tumor cell apoptosis warrant further in-depth exploration. In this study, we focused on mining small-molecule drugs that may directly target the PANoptosome. We analyzed the correlation between the mRNA expression of each PANoptosome gene and the half-maximal inhibitory concentration (IC_50_) of each small-molecule compound in two drug-related databases (GDCS and CTRP). Figure 8A, B summarize the correlation between gene expression and sensitivity to GDSC and CTRP drugs (top 30) in pan-cancer analysis. These results showed that the IC_50_ values of most of the top 30 drugs were significantly negatively correlated with the expression of RIPK3, CASP8, NLRP3, PYCARD, and CASP1, which implied that these small-molecule drugs might be potential activators of the PANoptosome. Notably, four small-molecule drugs, PIK-93, SNX-2112, AZD7762, and selumetinib, showed a significant correlation with the PANoptosome components in both the GDSC and CTRP databases (Fig. 8A, B). Among these, SNX-2112 and AZD7762 induce apoptosis in several tumor cells (Landau et al. 2012; Cheng et al. 2021; Ozgiray et al. 2022). To further evaluate the binding affinities of PIK-93, SNX-2112, AZD7762, and selumetinib for each PANoptosome component, molecular docking analysis was performed. The binding poses of the four candidate drugs and their corresponding interactions with protein residues of NLRP3, RIPK3, RIPK1, CASP6, CASP8, and CASP1, as well as the corresponding binding energy for each interaction, were obtained using Autodock Vina v.1.2.2 (Figs. 9, S8 and Table S8). We found that each candidate primarily bonded to these protein residues via hydrogen bonds and electrostatic interactions. In addition, the majority of these binding energies were less than − 5 kcal/mol, indicating stable binding between the four small-molecule drugs and the PANoptosome components. Among these, SNX-2112 and AZD7762 showed significantly stable binding with NLRP3 (Fig. 9A and G) and RIPK3 (Fig. 9B and H), with corresponding binding energies of less than − 8 kcal/mol. These findings provide insights into the mechanism of PANoptosome activation and its value in clinical applications.

## Discussion

In this study, we scrutinized the genetic, epigenetic, and transcriptional profiles of eight core PANoptosome component genes across 33 different cancers. A pronounced divergence was noted in the molecular characteristics of PANoptosome component genes between tumors and normal tissues. Certain PANoptosome genes exhibited a heightened SNV frequency and showed significant correlations with CNV and methylation. Furthermore, these genetic mutations and epigenetic modifications had a discernible impact on the expression of PANoptosome genes. This underscores the pivotal role of genetic alterations in driving cancer progression. Consequently, these findings not only lay the groundwork for an in-depth exploration of PANoptosome-related mechanisms but also enhance our comprehension of the intricacies associated with the participation of PANoptosome in tumorigenesis and development.

Building on the expression profiles of PANoptosome component genes, we devised PANo-RPI for evaluating the intrinsic PANoptosome assembly potential in cancers. Our observations revealed that the majority of cancers exhibited elevated PANo-RPI levels, particularly in MESO, LAML, DLBC, and THYM. Moreover, numerous cancers, including BRCA, CHOL, ESCA, HNSC, KIRC, KIRP, STAD, THCA, BLCA, and CESC, displayed higher PANo-RPI levels than their non-tumor counterparts. In parallel, we established a correlation between high PANo-RPI and improved prognosis in patients with ACC, BRCA, KICH, KIRP, MESO, SARC, READ, STAD, THCA, SKCM, and UVM. These findings suggest that, in comparison to normal cells, tumor cells exhibit increased intrinsic PANoptosis, while extrinsic PANoptosis is more likely triggered by external stimuli such as clinical treatments. However, a high PANo-RPI was associated with an unfavorable outcome in patients with HNSC, GBM, KIRC, LAML, LUAD, LGG, LUSC, OV, PAAD, and TGCT. The rapid and uncontrolled proliferation of cancer cells, coupled with their tendency to evade cell death, may offer an explanation for these contradictory results (Koo et al. 2015; Reina-Campos et al. 2018).

Targeting genes or pathways associated with the inflammatory response and reshaping the tumor microenvironment (TME) from a “cold” to a “hot” state holds promise as a strategy to enhance the effectiveness of immunotherapy (Duan et al. 2020; Wei et al. 2022). Our results demonstrated that a high PANo-RPI correlates with increased immune cell infiltration in the TME, including T cells, NK cells, macrophages, DCs, and others. Furthermore, elevated expression of immune checkpoints and enhanced antigen processing was consistently associated with high PANo-RPI in the majority of cancers. These findings suggest that patients with higher PANo-RPI may be more suitable candidates for immunotherapy. Additionally, through further analysis of PANo-RPI levels in various immunotherapy cohorts, we observed that responders to immunotherapy exhibited higher PANo-API than non-responders. Moreover, patients with higher PANo-RPI demonstrated significantly improved immunotherapy efficacy.CD8+cytotoxic T cells are key effector cells in anti-tumor immunity and immunotherapy (Pang et al. 2023). Our results revealed a positive association between PANo-RPI and the infiltration of CD8+T cells across all immunotherapy datasets. Collectively, these findings suggest that targeting PANoptosis in cancer cells could be an effective strategy for reshaping the “cold” TME into a “hot” one, thereby enhancing anti-tumor immune effects. Current treatment trends involve combination immunotherapies, such as the combination of chemotherapy with immune checkpoint inhibitor therapy (Chen et al. 2020). As an immunogenic cell death pathway, PANoptosis plays a crucial role in anti-tumor immunity and tumor suppression (Liu et al. 2022). However, the mechanisms facilitating tumor cell PANoptosis remain poorly understood. In this study, we identified some small-molecule drugs that may induce PANoptosis, such as SNX-2112 and AZD7762. These two drugs have previously been reported to induce apoptosis (Okawa et al. 2009; Wang et al. 2018; Hu et al. 2019). Consequently, SNX-2112 and AZD7762 will be employed in follow-up experiments to further explore their association with PANoptosis and assess their clinical applications in cancer treatments.

Certain types of precision medicine approaches have the potential to directly induce PANoptosis, effectively eliminating tumor cells through cytotoxic effects while bolstering the anti-tumor immune response. However, the application of targeted PANoptosis therapy, especially when combined with immunotherapy strategies like immune checkpoint inhibitors, may induce acute inflammation in any organ system, with pneumonitis representing one of the most severe immune-related adverse events (irAEs) (Spagnolo et al. 2022). In addition to short-term adverse events, there are lingering concerns regarding long-term adverse events resulting from therapies, such as idiopathic pulmonary fibrosis triggered by changes in immunologic and environmental factors (Karampitsakos et al. 2023a, b). Therefore, the simultaneous goal of maximizing the effectiveness of PANoptosis-based approaches while minimizing adverse events is a crucial consideration for the future development of new cancer treatment strategies.

## Supplementary Information

Below is the link to the electronic supplementary material.

Supplementary file1 (TIF 15812 kb)

Supplementary file2 (TIF 10570 kb)

Supplementary file3 (TIF 15349 kb)

Supplementary file4 (TIF 14732 kb)

Supplementary file5 (TIF 12548 kb)

Supplementary file6 (TIF 15485 kb)

Supplementary file7 (TIF 15504 kb)

Supplementary file8 (TIF 12726 kb)

Supplementary file9 (DOCX 23 kb)

Supplementary file10 (XLSX 122 kb)

## Data Availability

The data underling this article have been made available in the Cancer Genome Atlas (TCGA, http://cancergenome.nih.gov/) and Genotype-Tissue Expression (GTEx, http://commonfund.nih.gov/GTEx/) databases, and the corresponding clinical data were downloaded from the UCSC Xena (https://xenabrowser.net/datapages/). The gene expression matrices and clinical data of the immunotherapy cohorts were downloaded from the BioProject and GEO datasets at NCBI. The relevant data access details are as follows: PRJEB23709(https://www.ncbi.nlm.nih.gov/bioproject/PRJEB23709); GSE91061(https://www.ncbi.nlm.nih.gov/geo/query/acc.cgi?acc=GSE91061); GSE100797(https://www.ncbi.nlm.nih.gov/bioproject/?term=GSE100797); GSE35640 (https://www.ncbi.nlm.nih.gov/geo/query/acc.cgi?acc=GSE35640).
