# Supplementary figures and images for: Comprehensive Analyses of PANoptosome with Potential Implications in Cancer Prognosis and Immunotherapy
Source: Biochem Genet. 2024 Mar 4;63(1):331–53. doi: 10.1007/s10528-024-10687-8 (PMC11832696; doi:10.1007/s10528-024-10687-8)

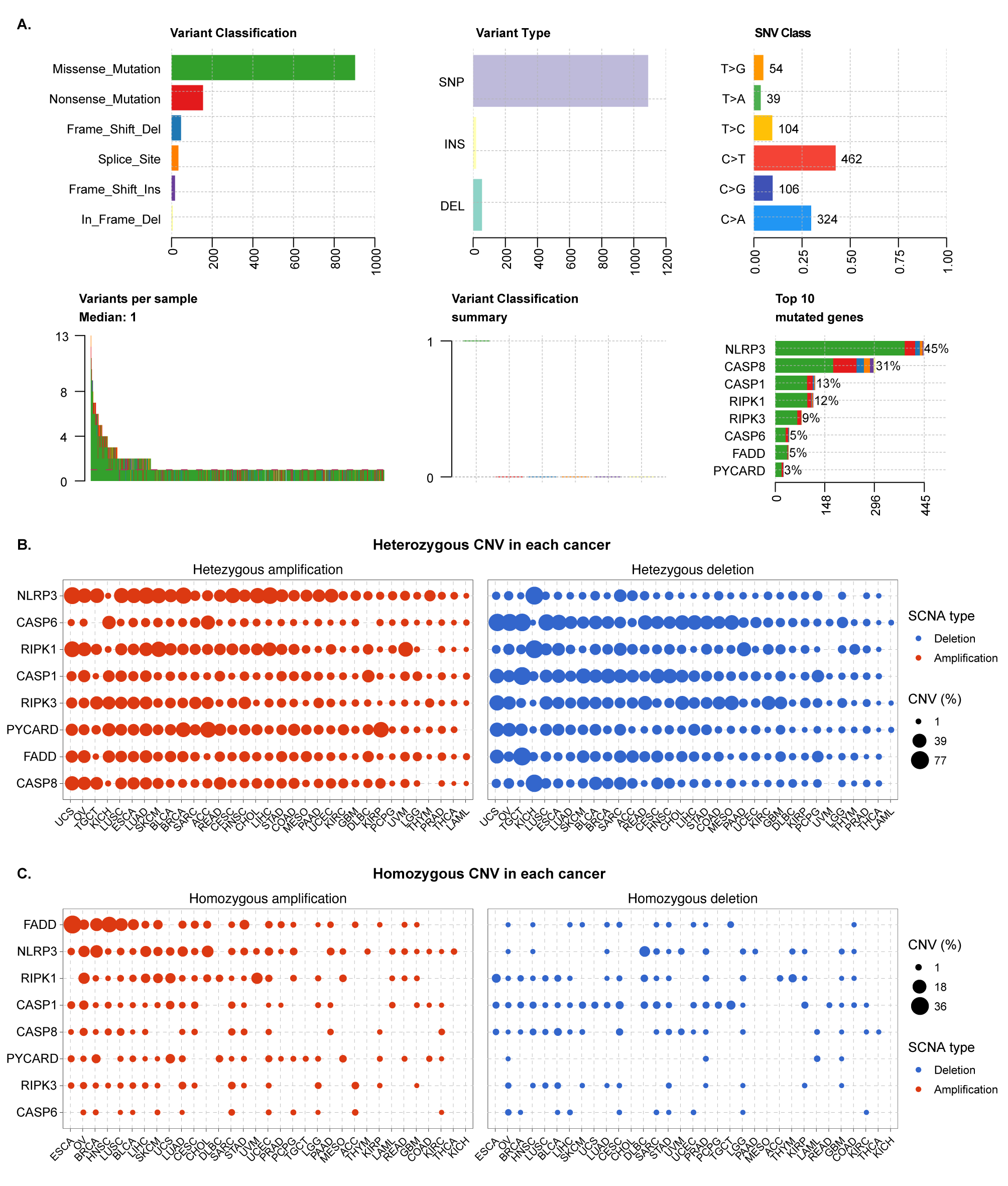

Supplement: Supplementary file 1 — Supplementary file1 (TIF 15812 kb) [file 10528_2024_10687_MOESM1_ESM.tif]

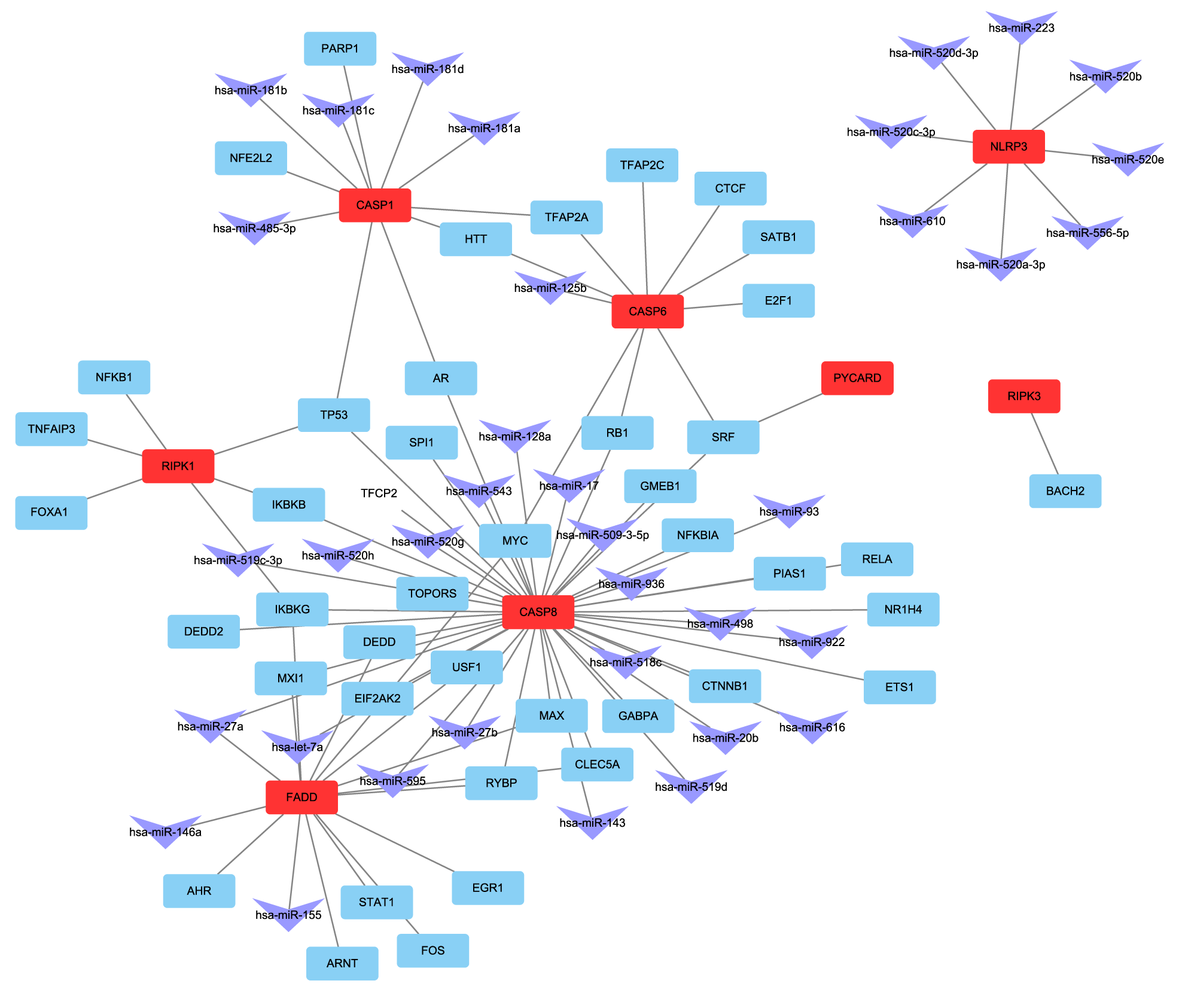

Supplement: Supplementary file 2 — Supplementary file2 (TIF 10570 kb) [file 10528_2024_10687_MOESM2_ESM.tif]

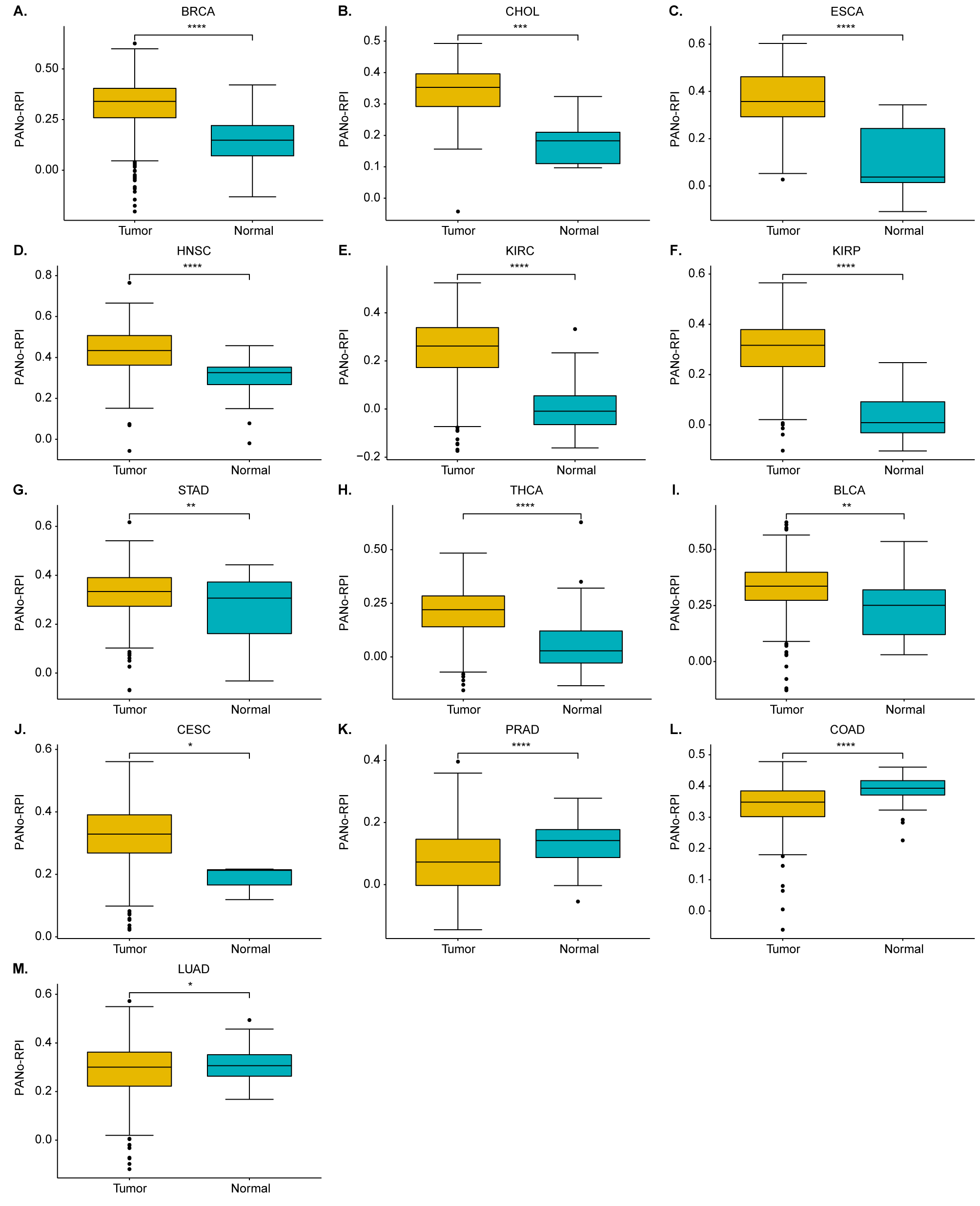

Supplement: Supplementary file 3 — Supplementary file3 (TIF 15349 kb) [file 10528_2024_10687_MOESM3_ESM.tif]

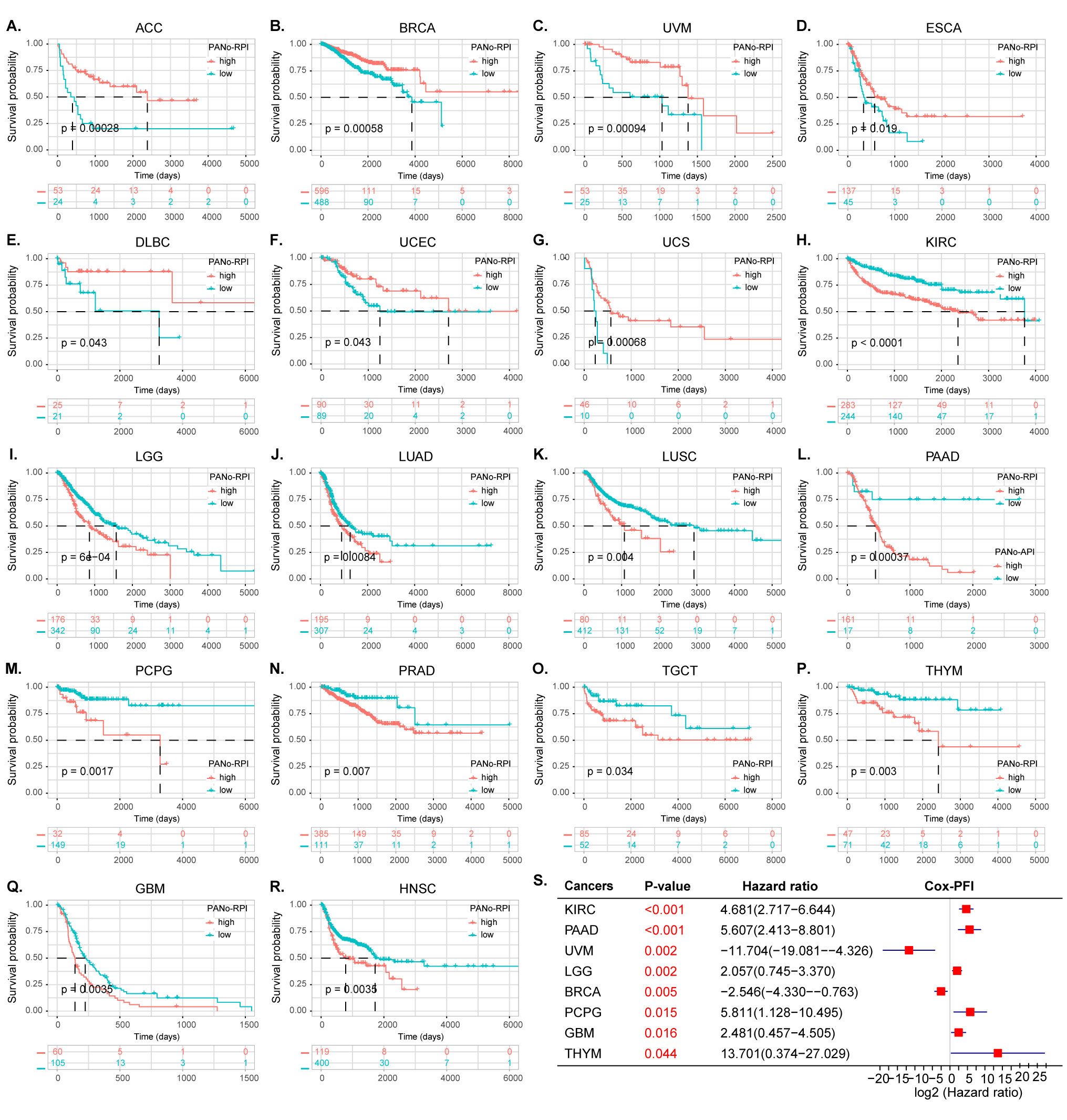

Supplement: Supplementary file 4 — Supplementary file4 (TIF 14732 kb) [file 10528_2024_10687_MOESM4_ESM.tif]

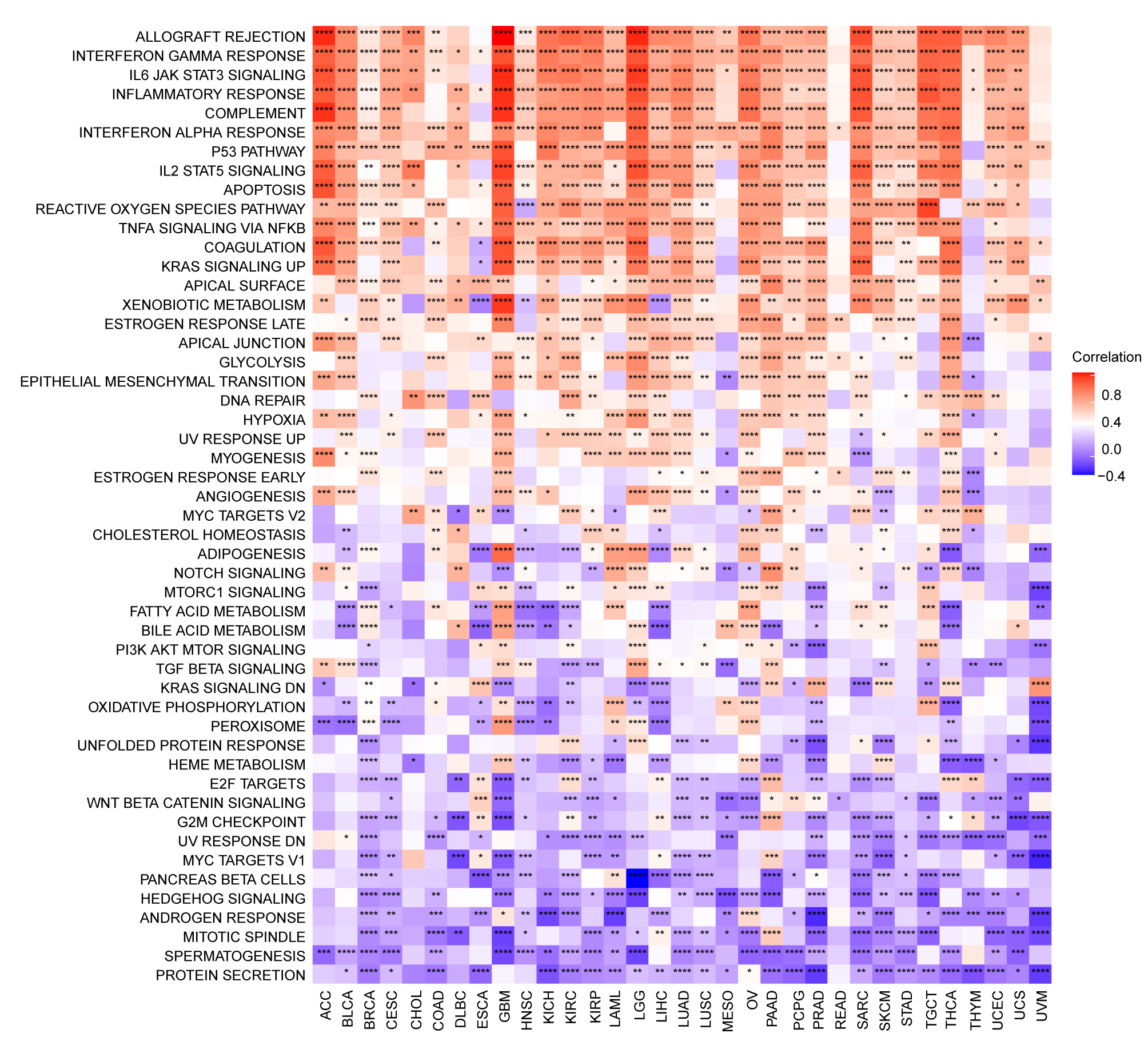

Supplement: Supplementary file 5 — Supplementary file5 (TIF 12548 kb) [file 10528_2024_10687_MOESM5_ESM.tif]

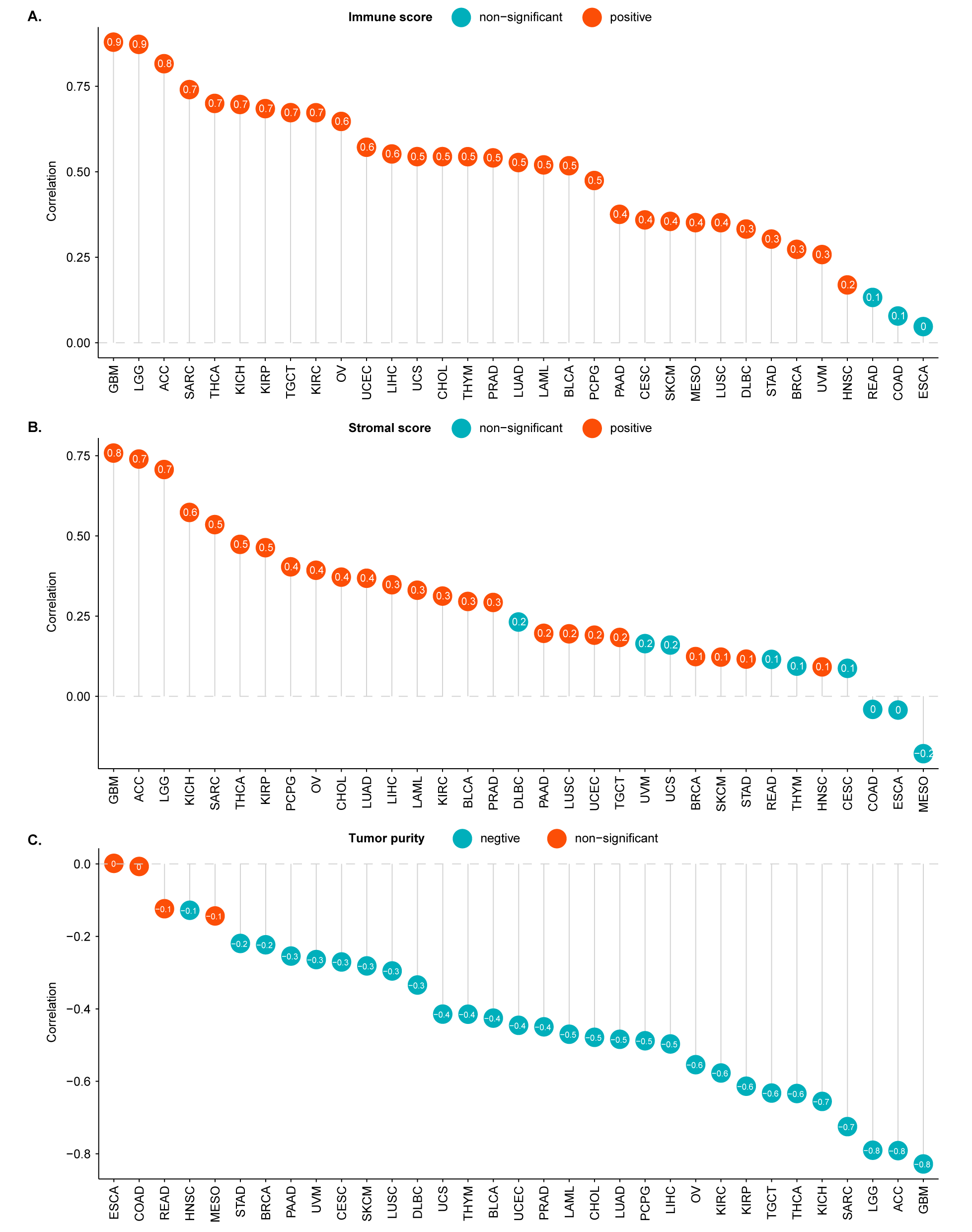

Supplement: Supplementary file 6 — Supplementary file6 (TIF 15485 kb) [file 10528_2024_10687_MOESM6_ESM.tif]

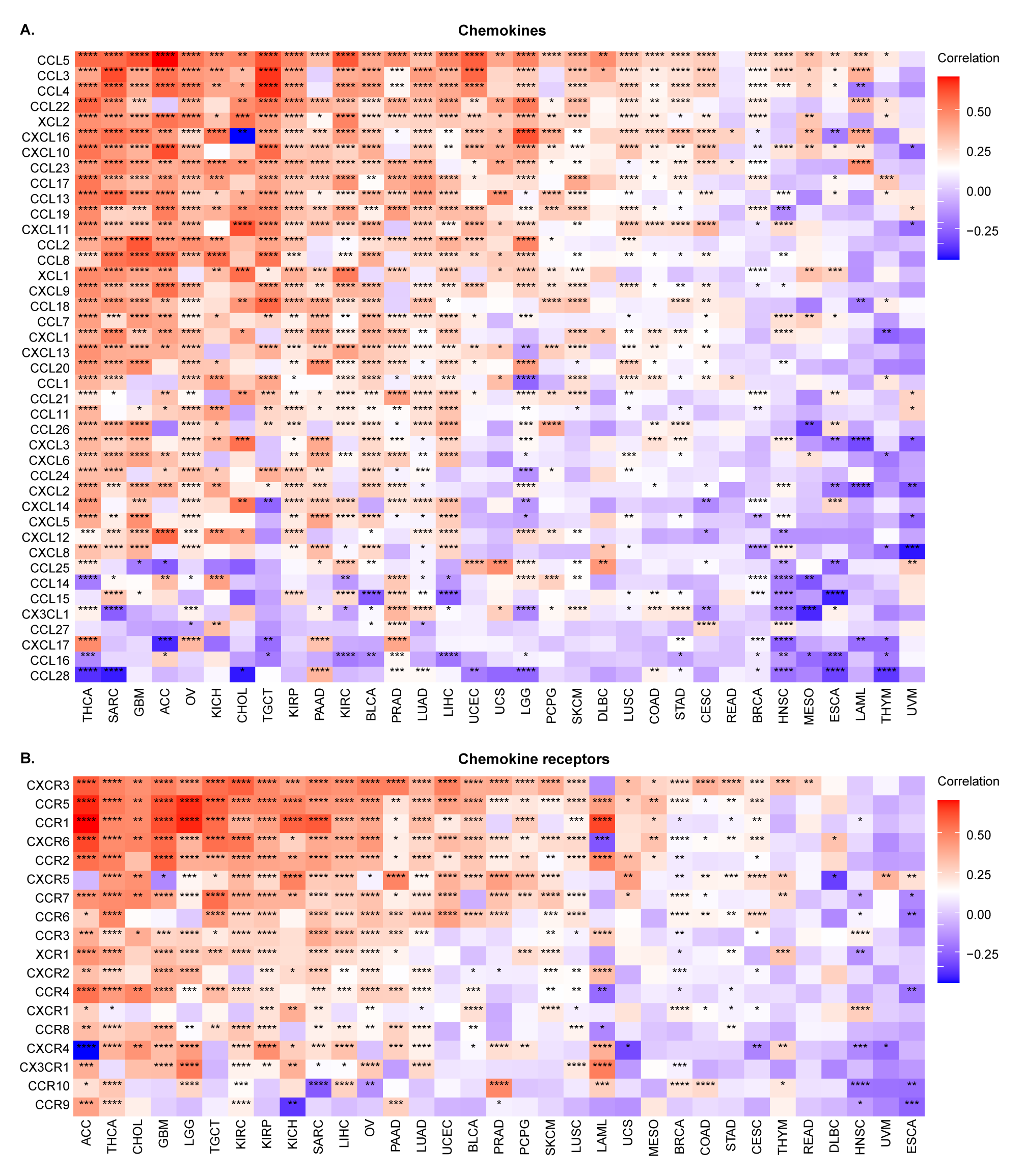

Supplement: Supplementary file 7 — Supplementary file7 (TIF 15504 kb) [file 10528_2024_10687_MOESM7_ESM.tif]

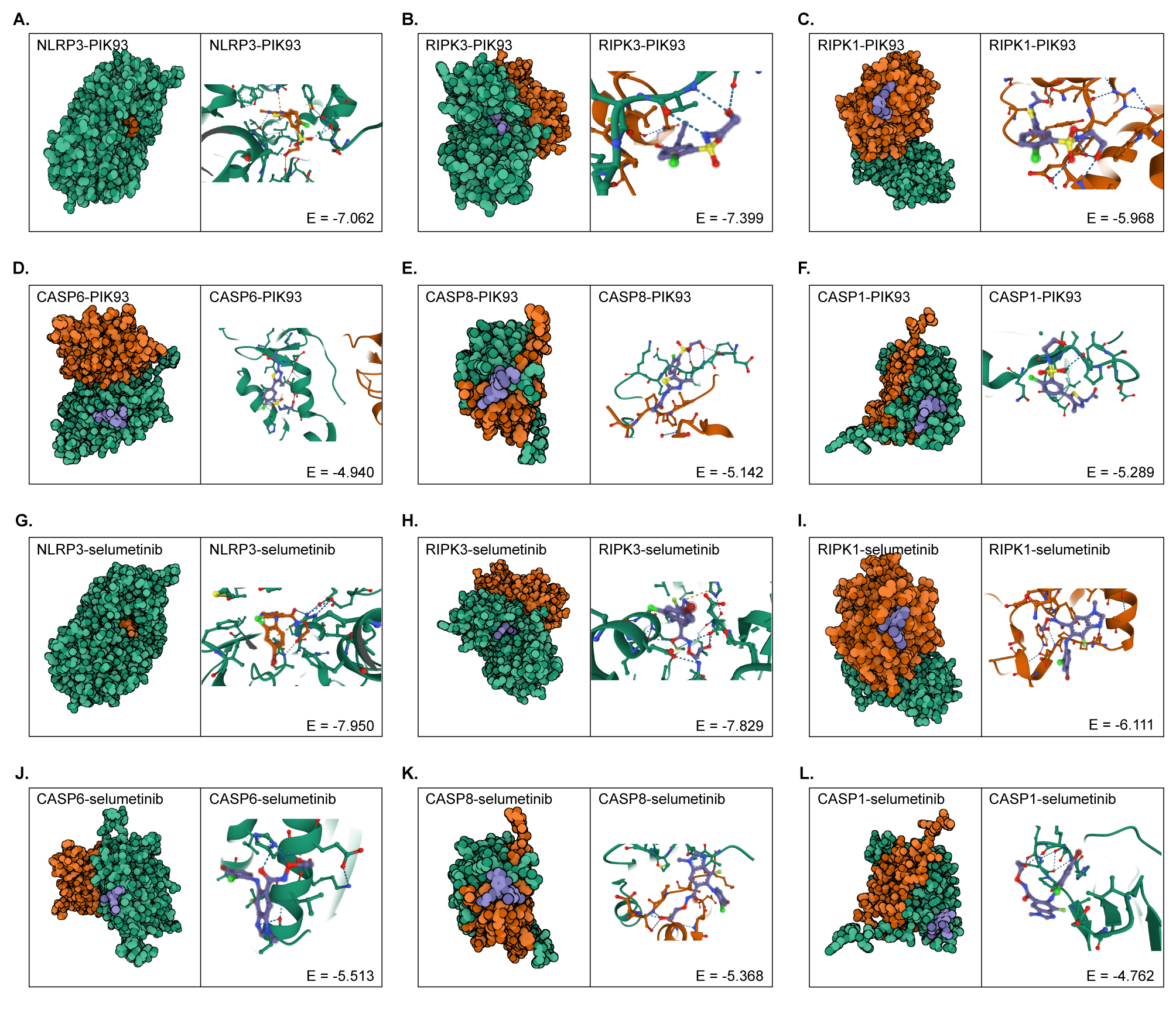

Supplement: Supplementary file 8 — Supplementary file8 (TIF 12726 kb) [file 10528_2024_10687_MOESM8_ESM.tif]
