# Supplementary material for: Comprehensive Analyses of PANoptosome with Potential Implications in Cancer Prognosis and Immunotherapy
Source: Biochem Genet. 2024 Mar 4;63(1):331–53. doi: 10.1007/s10528-024-10687-8 (PMC11832696; doi:10.1007/s10528-024-10687-8)
Supplement: Supplementary file 9 — Supplementary file9 (DOCX 23 kb) [file 10528_2024_10687_MOESM9_ESM.docx]

Supplementary Materials

# Supplementary Figures

**Fig. S1** (**A**) The single nucleotide variation (SNV) classes of PANoptosome-related genes across 33 cancers. (**B**) The profile of heterozygous CNV of PANoptosome-related genes. **(C)** The profile of homozygous CNV of PANoptosome-related genes.

**Fig. S2** Interaction network of transcription factors (TFs)-microRNAs (miRNAs)-PANoptosome component genes.

**Fig. S3** Box plots illustrating the comparison of levels between tumors and non-tumor tissues.

**Fig. S4** (**A-R)** Kaplan-Meier progression-free interval (PFI) curves stratified by high and low PANo-RPI in cancers. (**S**) Forest plot depicting the results of univariate Cox regression analyses for PFI associated with PANo-RPI across various cancer types.

**Fig. S5** Heatmap illustrating the correlation of PANo-RPI with the enrichment scores of hallmark pathways.

**Fig. S6** Lollipop plots illustrating the correlation of PANo-RPI with **(A)** immune scores, **(B)** stromal scores, and **(C)** tumor purity.

**Fig. S7** Heatmap displaying the correlation of PANo-RPI with **(A)** chemokines and **(B)** chemokine receptors.

**Fig. S8 (A-F)** Molecular docking simulations of PIK-93 with (**A**) NLRP3, (**B**) RIPK3, (**C**) RIPK1, (**D**) CASP6, (**E**) CASP8 and (**F**) CASP1. **(A-F)** Molecular docking simulations of selumetinib with (**G**) NLRP3, (**H**) RIPK3, (**I**) RIPK1, (**J**) CASP6, (**K**) CASP8 and (**L**) CASP1.

# Supplementary Tables

**Table. S1** Abbreviations of 33 cancer types.

**Table. S2** Results of univariate Cox regression analysis for overall survival of PANoptosome-related genes.

**Table. S3** Gene Set Variation Analysis (GSVA) results of hallmark gene sets (h.all.v7.4.symbols.gmt).

**Table. S4** Results of the correlation analysis between PANo-RPI and the enrichment scores of immune-related signatures from Mariathasan et al.

**Table. S5** Results of the correlation analysis between PANo-RPI and tumor immune scores, stromal scores, and purity.

**Table. S6** Results of the correlation analysis between PANo-RPI and the infiltration of immune cells into tumors.

**Table. S7** Results of the correlation analysis between PANo-RPI and TMB scores.

**Table. S8** Results of the correlation analysis between PANo-RPI and MSI scores.

**Table. S9** Binding energies between PANoptosome components and small-molecule drugs.
